# Critical dialogue: Anterior cruciate ligament reconstruction: evolving surgical concepts and their radiological implications

**DOI:** 10.1186/s13244-026-02358-6

**Published:** 2026-08-06

**Authors:** Emre Anıl Özbek, Zehra Akkaya

**Affiliations:** 1 Department of Orthopaedic Surgery, Ankara University, Ankara, Turkey; 2 Department of Radiology, Ankara University, Ankara, Turkey

## Current practice and knowledge

Anterior cruciate ligament (ACL) injuries are among the most common ligamentous injuries of the knee [1–3]. As surgical techniques continue to evolve, the role of the radiologist extends beyond simple confirmation of ACL injury, with growing emphasis on comprehensive MRI interpretation relevant to clinical assessment and surgical evaluation [4].

ACL injury is primarily diagnosed through patient history and targeted physical examination, while MRI remains the most sensitive and specific imaging modality for confirmation [5–7]. In tertiary musculoskeletal centers, comprehensive MRI interpretation is well established. However, an increasing number of patients undergo MRI at outside institutions. In such settings, technical limitations, suboptimal imaging parameters, and workflow constraints may limit comprehensive radiological assessment. In inconspicuous cases, additional images acquired in sagittal oblique (parallel to the lateral femoral condyle) and coronal-oblique (along the ACL axis, from the roof of the intercondylar notch) planes, or isotropic volumetric 3D images (≤ 2.5 mm; DESS, SPACE) may aid in comprehensive knee evaluation, which may not always be feasible [8]. Nevertheless, even within practical limitations, radiology reports should aim to include as much clinically relevant information as reasonably achievable beyond isolated confirmation of ACL disruption.

The true clinical value of MRI in this setting lies not only in diagnostic confirmation but also in providing additional information that may support preoperative assessment and surgical evaluation. Radiological assessment should therefore extend beyond ligament disruption to include evaluation of notch morphology, tibial slope, associated intra- and extra-articular pathology, meniscal ramp lesions, anterolateral ligament abnormalities, and anatomical features relevant to surgical strategy, such as the thickness and length of potential autologous graft donor sites.

Ideally, MRI findings should be interpreted within a multidisciplinary framework involving close collaboration between the radiologist and the orthopedic surgeon. However, such direct interdisciplinary interaction may not always be feasible across all clinical settings. Consequently, detailed and treatment-oriented radiology reporting becomes particularly important, as comprehensive reporting may provide clinically actionable information that facilitates surgical planning, reduces the need for repeated imaging and repeated expert interpretation, and contributes to more efficient and individualized patient management, even in the absence of direct collaborative discussion.

## Recent advancements

The orthopedic management of ACL injury has evolved in parallel with advances in the understanding of knee biomechanics. Early surgical efforts, began with primary ACL repair in 1895 and were followed by the first reconstruction in 1917 [9]. With the adoption of arthroscopic techniques, transtibial single-bundle reconstruction became widely used; however, later studies showed that this approach often resulted in non-anatomical femoral tunnel placement and inadequate control of rotational instability. In response, double-bundle reconstruction was introduced to better replicate native ACL anatomy, although long-term clinical superiority over refined single-bundle techniques has not been consistently demonstrated [9, 10].

Contemporary ACL reconstruction reflects a shift toward individualized, biomechanically driven strategies. Alongside renewed attention to graft selection—historically dominated by hamstring tendon and bone–patellar tendon–bone autografts—quadriceps tendon autografts have gained increasing popularity due to their favorable graft size, strength, and reliable clinical outcomes [11]. Moreover, growing recognition that isolated intra-articular reconstruction may be insufficient in patients with significant rotational instability has led to increased use of lateral extra-articular procedures, such as lateral extra-articular tenodesis and anterolateral ligament reconstruction, particularly in professional athletes, patients with high-grade pivot shift, generalized ligamentous laxity, and revision surgeries [1, 12].

Recent recognition of the knee–brain axis has further expanded the understanding of ACL injury beyond isolated mechanical instability. Disruption of proprioceptive input and arthrogenic muscle inhibition, particularly affecting quadriceps function, may persist despite anatomically successful reconstruction and contribute to ongoing functional deficits [13, 14].

These concepts highlight the importance of comprehensive radiological evaluation in supporting patient-specific surgical strategies and assessment of rotational stability.

## Impact on radiology practice and research

Advances in ACL surgery and evolving biomechanical concepts require a corresponding change in radiological practice. MRI assessment should include evaluation of associated intra- and extra-articular pathology, particularly in revision cases where failure mechanisms are often multifactorial [15, 16]. Assessment of bone and cartilage status, notch morphology, meniscal lesions, posterior tibial slope, and anterolateral structures contributes to integrated radiological evaluation and individualized surgical assessment.

Furthermore, the increasing number of revision ACL procedures highlights the need for radiologists to be familiar with postoperative and revision-specific imaging findings [3]. These include evaluation of tunnel position and widening, graft integrity and ligamentization, graft rupture or stretching, cyclops lesions, fixation-related complications, and residual or untreated rotational instability [14]. Although comprehensive assessment may not always be feasible in all clinical settings because of variable MRI quality, metal-related artifacts, or technical limitations, radiology reports should still aim to include as much clinically relevant information as reasonably achievable. In cases where tunnel widening or complex bony anatomy is suspected, computed tomography (CT) could play a complementary role as it provides superior assessment of tunnel morphology and bone stock for revision planning. Furthermore, potential graft donor sites should be carefully assessed for pre-existing abnormalities that may affect graft selection or revision strategy [17].

Quantitative MRI techniques, including T1ρ and T2 mapping, diffusion imaging, compositional cartilage imaging, isotropic 3D sequences, and emerging ultra-high-field 7-T MRI applications, have shown promising results for the evaluation of graft maturation, cartilage integrity, and periarticular soft tissues [18, 19]. Although many of these techniques are currently more commonly used in advanced tertiary or research-oriented centers, ongoing technological developments and increasing availability may contribute to more comprehensive ACL assessment in future clinical practice. Consistent, collaborative, and clinically focused imaging interpretation remains essential for reducing variability in care and supporting the effective application of evolving surgical protocols across different clinical settings.

## Data Availability

Not applicable.
